# Factors influencing the decision to discontinue treatment due to chemotherapy-induced peripheral neuropathy among patients with metastatic breast cancer: a best–worst scaling

**DOI:** 10.1007/s00520-025-09508-4

**Published:** 2025-05-10

**Authors:** Rotana M. Radwan, John F. P. Bridges, Daniel L. Hertz, Maryam B. Lustberg, Hetal Vachhani, Erin Hickey Zacholski, Vanessa B. Sheppard, Teresa M. Salgado

**Affiliations:** 1 Department of Pharmacotherapy & Outcomes Science, School of Pharmacy, Virginia Commonwealth University, 410 N 12 Street, PO Box 98053, Richmond, VA 23298 USA; 2 Department of Biomedical Informatics, College of Medicine, The Ohio State University Wexner Medical Center, 250 Lincoln Tower, 1800 Cannon Drive, Columbus, OH 43210 USA; 3 Department of Clinical Pharmacy, College of Pharmacy, University of Michigan, 428 Church St, Ann Arbor, MI 48109 USA; 4 Department of Medical Oncology, School of Medicine, Yale University, 20 York St, New Haven, CT 06510 USA; 5 Department of Internal Medicine, School of Medicine, Virginia Commonwealth University, VCU Medical Center, 1201 E Marshall St #4-100, Richmond, VA 23298 USA; 6 Department of Social and Behavioral Sciences, School of Public Health, Virginia Commonwealth University, 830 E Main Street, Richmond, VA 23219 USA; 7 Massey Comprehensive Cancer Center, Virginia Commonwealth University, 401 College St, Richmond, VA 23298 USA

**Keywords:** Patient preferences, Best–worst scaling, Chemotherapy-induced peripheral neuropathy, Metastatic breast cancer

## Abstract

**Purpose:**

To measure the importance of factors that influence the decision to discontinue treatment due to chemotherapy-induced peripheral neuropathy (CIPN) among patients with metastatic breast cancer (mBC).

**Methods:**

An online survey incorporating a best–worst scaling (BWS) was conducted among women in the USA with mBC and experiencing CIPN. In the BWS, women chose the most and least important factors influencing their decision to discontinue treatment due to CIPN. Seven factors were included: relieving current neuropathy symptoms, reducing risk of long-term neuropathy, having another cancer treatment option, understanding the risk of treatment discontinuation, and receiving support for treatment discontinuation from the oncologist, loved ones, or patients with similar experiences. To measure the importance of each factor, a conditional logit model estimated coefficients, which were subsequently rescaled to importance scores that summed to 100. The dependent variable was the choice of a factor as most or least important across seven choice tasks.

**Results:**

The sample included 189 women with a mean age of 52.5 (SD = 12.65) years, 52.9% were White, 33.9% were Black, and 64.6% held a bachelor’s degree or higher. When faced with the decision to discontinue treatment due to CIPN, the most important factors were having another cancer treatment option (score 23.5), followed by understanding the risk of treatment discontinuation (score 19.2), and reducing risk of long-term neuropathy (score 19.1). The least important factors in the decision to discontinue treatment due to CIPN were: support from loved ones (score 5.2) and support from other patients (score 3.3).

**Conclusion:**

When faced with the decision to discontinue treatment due to CIPN, women with mBC attributed more importance to survival and reducing the risk of long-term CIPN. Knowledge of what matters most to patients may assist with shared decision-making to optimize therapeutic outcomes in patients receiving neurotoxic chemotherapy.

**Supplementary Information:**

The online version contains supplementary material available at 10.1007/s00520-025-09508-4.

## Background

Chemotherapy-induced peripheral neuropathy (CIPN) is a debilitating side effect of neurotoxic chemotherapy agents that affects over 60% of patients with metastatic breast cancer (mBC) [1–3]. Women with breast cancer describe CIPN as one of the most distressing treatment-related symptoms and a major cause of deterioration in function and of low quality of life [4]. CIPN affects the nerves responsible for sensation and movement, causing numbness, tingling, thermal sensitivity, pain, and muscle weakness [2, 5]. Symptoms often start in the hands and feet and move inward in a “glove and stocking” distribution, impacting patients’ ability to perform activities of daily living [2, 5]. These negative consequences can persist for several months or even years after the completion of neurotoxic chemotherapy, and in some cases, they may become permanent [6, 7].

Few therapeutic options are available to prevent or treat CIPN, and existing treatments have limited effectiveness [8, 9]. The American Society of Clinical Oncology (ASCO) guidelines recommend that clinicians assess and discuss the appropriateness of discontinuing chemotherapy with patients who develop intolerable CIPN [8]. Early treatment discontinuation decreases the relative dose intensity and the total administered dose, and may compromise treatment effectiveness (i.e., progression-free survival) [9–11]. Consequently, the decision to discontinue treatment requires balancing the risk of compromising treatment effectiveness and the potential benefit of mitigating CIPN progression [10, 12]. Recommendations guiding when to discontinue chemotherapy according to CIPN severity may be unclear or variably applied, contributing to decision-making uncertainty [8, 11]. Under these circumstances, it becomes important to incorporate patient priorities into the decision-making process. Eliciting patient priorities facilitates more personalized health care that aligns with patients’ desired outcomes [13]. Additionally, knowledge of patients’ priorities enables tailoring information provision and prevents information overload, which can occur when summarizing all treatment outcome possibilities during shared decision-making [14, 15].

While it remains unknown how patients with CIPN prioritize different factors influencing their decision to discontinue treatment, several qualitative studies provide insights into the factors involved. Patients report tolerating short-term CIPN discomfort to prevent potential cancer recurrence, while others endure CIPN believing that the symptoms will be reversible [16]. Those who have experienced long-term CIPN expressed that they would have asked to discontinue treatment early had they known that it was an option [17]. Many patients rely on clinicians, and occasionally family members, to determine the level of CIPN they should endure to achieve treatment benefits [16]. Others seek information about CIPN from patient forums where cancer survivors share experiences and strategies for managing symptoms [16]. However, those who are well-informed about their condition tend to trust their healthcare providers and feel involved in the decision-making process of CIPN management, highlighting the critical role of patient-clinician communication [18].

Despite valuable insights from qualitative research, these studies do not quantify the order in which patients prioritize various factors (e.g., survival, quality of life, or psychosocial considerations) affecting the decision to discontinue treatment due to CIPN and their importance relative to each other. Thus, this study sought to measure the factors identified by patients with mBC as most and least important when deciding to discontinue treatment due to CIPN. The findings will improve patient-clinician communication by offering valuable information into the patient’s perspective and contribute to patient-centered care.

## Methods

### Study design

A best–worst scaling (BWS) exercise to elicit respondents’ attributed importance to various factors that influence the decision to discontinue treatment due to CIPN was developed and included in a survey [19–21]. In a BWS, respondents choose the best (most important) and worst (least important) among a subset of influential factors (i.e., choice task). Respondents complete multiple BWS choice tasks, each presenting a different combination of factors, and the resulting choices are analyzed together to estimate the importance of the factors [22, 23]. This study was conducted according to good research practices for conjoint analysis by the International Society for Pharmacoeconomics and Outcomes Research (ISPOR) [24] and a patient-centered framework for instrument development [25]. The study was determined exempt by the Virginia Commonwealth University (VCU) Institutional Review Board.

### Sample and recruitment

Eligible respondents were adult women with mBC who were currently receiving or had previously received neurotoxic treatment (i.e., completed or discontinued) and who had prior or current experience of CIPN. This broad eligibility criterion ensured the inclusion of diverse perspectives, capturing the variability in treatment decision-making influenced by CIPN across different stages of care and varying symptom severity levels. Respondents also had to speak and read English and reside in the USA.

A multimodal recruitment strategy was implemented for all study phases, including stakeholder engagement, pretesting, and survey dissemination. Recruitment efforts comprised posting flyers on breast cancer social media support groups, featuring study materials in newsletters disseminated by breast cancer organizations, and encouraging word-of-mouth referrals among patients. Eligible patients were also identified with the assistance of the Cancer Informatics Core from the VCU Massey Comprehensive Cancer Center. Patients were invited to participate via mailed letters, with up to three follow-up phone calls and one reminder email to encourage participation and increase response rate. Interested individuals contacted the lead author (RR) to participate and received a personalized survey link via email. The overlapping recruitment approaches made determining a response rate challenging. Additionally, a convenience sample of oncology clinicians was recruited via email from the VCU Massey Comprehensive Cancer Center for consultation during instrument development. Clinicians were offered $25 compensation; however, two of the three declined payment. Patients received a $50 gift card as a token of appreciation for their time.

### Instrument development

The development of the BWS instrument used a stakeholder-driven, iterative approach, engaging three oncology clinicians—two breast medical oncologists and one oncology pharmacist—and seven patients with mBC who had experienced CIPN. The first step involved a thorough review of the literature to identify factors influencing the decision to discontinue treatment due to CIPN. This review covered qualitative studies that explored patients lived experiences with CIPN [4, 16–18, 26–29], guidelines on CIPN management [6, 8, 11, 30–32], CIPN assessment measures [33–36], observational studies [2, 37, 38], and clinical trial data [11, 38, 39]. The identified factors (*n* = 12) were then presented to clinicians and patient stakeholders in separate virtual meetings, either individually or in groups. Clinicians evaluated the clinical accuracy and relevance of the factors and were asked to suggest additional ones. Patients were asked to share their experiences, assess whether the factors resonated with them, and suggest additional ones. Following two individual patient interviews, two researchers iteratively revised the list of factors and their descriptions. Additional revisions were made during a group meeting with five patients to ensure accuracy and comprehensibility of factors and their respective descriptions. Meetings were audio-recorded and transcribed verbatim by a professional party to facilitate the compilation of feedback in a written format. Regular consultations with a patient preference expert (JFB) were held, and changes made as a result were discussed with patients for feedback and approval. After removing redundant or overlapping factors, the final instrument consisted of seven factors, categorized into two domains: clinical and psychosocial (Table 1). A BWS choice task was developed based on these seven factors and pretested with 20 participants using in-depth “think aloud” exercises to assess comprehension, terminology, willingness to tradeoff, and cognitive ease [40]. This process resulted in minor language modifications and added clarification as appropriate.

**Table 1:** Factors used in the best–worst scaling and their respective descriptions

Domain	Factor and description
Clinical	**Relieving current neuropathy symptoms** Treatment discontinuation can help relieve the neuropathy symptoms that you are currently experiencing
Clinical	**Reducing risk of long-term neuropathy** Treatment discontinuation can lower the risk of developing long-term neuropathy that lasts for years even after discontinuing the treatment that was causing neuropathy
Clinical	**Having another cancer treatment option** Having the option to switch to another cancer treatment that doesn’t cause neuropathy but still helps fight off cancer
Clinical	**Understanding the risk of treatment discontinuation** Understanding how treatment discontinuation can affect the growth of your cancer
Psychosocial	**My oncologist supports treatment discontinuation** Support from your oncologist to discontinue treatment
Psychosocial	**My loved ones support treatment discontinuation** Support from loved ones, such as family, friends, or partners to discontinue treatment
Psychosocial	**Patients like me support treatment discontinuation** Support from other patients who have undergone similar experiences and have made similar decisions

**Fig. 1:**
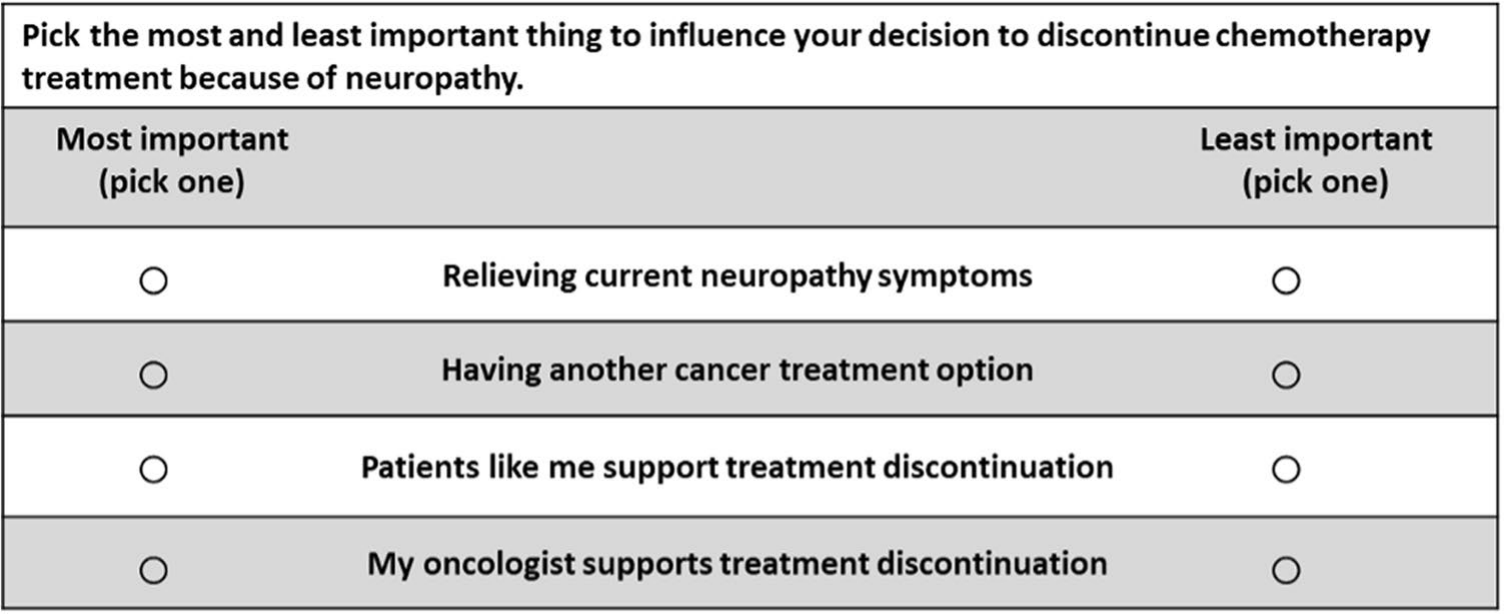
Example of a choice task

Preference elicitation was framed within a vignette in which respondents were instructed to picture themselves making a decision to discontinue treatment due to CIPN, alongside their oncologist, and the factors that would influence that decision. A Youden experimental design with seven factors was presented in seven subsets of four factors each. Respondents were asked to choose the most and the least important factors within each choice task (Fig. 1) to influence their decision to discontinue treatment due to CIPN. The choice tasks were constructed using a balanced incomplete block design, ensuring that each factor appeared with equal occurrence and co-occurrence of all other factors. Prior to starting the experiment, respondents were provided with training materials including an explanation and an example of how to complete a BWS choice task. Furthermore, respondents rated the importance of each factor on a three-point scale, as a means to familiarize themselves with the factors and ensure that they read and understood their descriptions before proceeding with the BWS (Appendix A).

### Data collection

The final survey was organized into the following sections: (1) patients’ clinical history, (2) the BWS, and (3) patients’ demographic characteristics (Appendix A). Before starting the survey, respondents answered screening questions to ensure that they met the eligibility criteria, followed by reading information about the study procedures and providing informed consent.

Clinical information collected in the first section of the survey included: years since mBC diagnosis, whether they were currently receiving treatment for mBC, years since CIPN onset, current experience of CIPN symptoms, and treatment adjustments due to CIPN, such as delay, reduction, or discontinuation. Neuropathy experience was assessed using the two-item PRO-CTCAE measure to assess the severity of numbness/tingling in the hands and feet (0 = none, 1 = mild, 2 = moderate, 3 = severe, 4 = very severe) and the degree to which numbness/tingling interfered with usual daily activities (0 = not all, 1 = a little bit, 2 = somewhat, 3 = quite a bit, 4 = very much) [35]. The PRO-CTCAE items use a recall reference period of “the past seven days,” as research has shown this timeframe to be reliable without a substantial loss of information [35, 41]. Demographic information provided after completing the BWS exercise included: age, race/ethnicity, educational attainment, income, and state where respondents resided.

The self-administered survey was anonymous and distributed using Qualtrics (Provo, Utah). The survey was open for 12 weeks, between July and September 2023, and took about 30 min to complete. Respondents did not have the option to skip any questions or choice tasks; therefore, there were no missing data.

### Statistical analysis

Descriptive statistics summarized the sample characteristics. A conditional logit analysis was conducted using a sequential best–worst assumption according to which respondents were assumed to have first selected the most important factor, followed by the least important factor in each choice task [42]. The BWS data were effect coded and analyzed using a conditional logit model [43]. The dependent variable was the choice of a factor as most or least important across seven choice tasks to estimate the importance associated with seven factors (independent variables) [42]. A coefficient *β* was estimated for each factor, with coefficients reflecting preference weights. Higher preference weight means that a factor is more prioritized, lower preference weight means that a factor is less prioritized [42]. Coefficients derived from the conditional logit analysis were probability rescaled to generate importance scores, simplifying interpretation. In this rescaling, the sum of the importance scores for all factors included in the BWS is 100, where higher scores indicate higher importance of the factor [44]. This transformation uses a ratio scale, where a factor with a score of 20 is deemed twice as important as one with a score of 10 [44]. If all BWS scores had equal importance, each factor would have an importance score of 14.3 (i.e., 100 divided by 7). Finally, ratings of each of the factors during the familiarization exercise were used as a secondary data source to compare and validate the BWS findings using Spearman’s rank test. Statistical tests were two-tailed at an alpha level of 0.05. All data were analyzed using Stata19 (StataCorp LP, College Station, TX).

## Results

A total of 189 women completed the survey (Table 2). Of these, 36.0% were recruited from VCU’s Massey Comprehensive Cancer Center and 64.0% from other recruitment channels (e.g., breast cancer organization newsletters and private breast cancer groups in social media platforms). Women were on average 52.5 (SD = 12.65) years, about half were White (52.9%), 64.6% held a bachelor’s degree or higher, and 32.8% had a household income of $85,000 or higher.

**Table 2:** Patient baseline characteristics

Characteristics	Sample (*n* = 189)
**Age, mean (SD)**	52.5 (12.65)
**Race/ethnicity, *n* (%)**	
White, non-Hispanic	100 (52.9)
Black, non-Hispanic	64 (33.9)
Hispanic	11 (5.82)
Other, non-Hispanic	14 (7.4)
^**a**^**Region, *n* (%)**	
South	117 (61.9)
Northeast	31 (16.4)
West	29 (15.3)
Midwest	18 (9.5)
**Education, *n* (%)**	
Bachelor’s degree or higher	122 (64.6)
Some college	57 (30.2)
High school or less	10 (5.3)
**Income, *n* (%)**	
Less than $34,999	38 (20.1)
$35,000–$84,999	60 (31.8)
More than $85,000	62 (32.8)
Prefer not to say	29 (15.3)
**Years since diagnosis, *n* (%)**	
Less than 1 year	21 (11.1)
Between 1 and 5 years	113 (59.8)
More than 5 years	55 (29.1)
**Years experiencing CIPN, *n* (%)**	
Less than 1 year	44 (24.3)
Between 1 and 5 years	96 (53.0)
More than 5 years	41 (22.7)
^**b**^**CIPN severity, *n* (%)**	
Numbness/tingling in hands and feet	
None	7 (3.70)
Mild	48 (25.4)
Moderate	96 (50.8)
Severe	28 (14.8)
Very severe	10 (5.3)
Interference with usual daily activities	
Not at all	27 (14.3)
A little bit	56 (29.6)
Somewhat	54 (28.6)
Quite a bit	36 (19.0)
Very much	16 (8.5)
**Adjusted treatment, *n* (%)**	
Yes	
**Type of treatment adjustment, *n* (%)**	
Delay	12 (15.4)
Reduce	27 (34.6)
Discontinue	23 (29.5)
Delay and reduce	9 (11.54)
Delay and discontinue	1 (1.3)
Reduce and discontinue	4 (5.13)
Delay, reduce, and discontinue	2 (2.6)

^a^Original variable collected as state of residence, which was grouped according to US census regions

^b^The two-item PRO-CTCAE measure evaluates the severity of numbness/tingling in the hands and feet (0 = none, 1 = mild, 2 = moderate, 3 = severe, 4 = very severe) and the degree to which numbness/tingling interfered with usual daily activities (0 = not at all, 1 = a little bit, 2 = somewhat, 3 = quite a bit, 4 = very much)

Over half of the women had been diagnosed with mBC for 1 to 5 years (59.8%) and started experiencing CIPN between 1 and 5 years ago (53.0%). Moderate numbness and tingling in the hands and feet was reported by 50.8% of respondents, while 29.6% indicated “a little bit” of interference with daily activities on the PRO-CTCAE sensory neuropathy scale. Among patients whose oncologists had recommended treatment adjustments (41.3%), the majority had a dose reduction (34.6%) or treatment discontinuation (29.5%).

The most important factors identified by respondents were: “Having another cancer treatment option” (score 23.5), “Understanding the risk of treatment discontinuation” (score 19.2), and “Reducing risk of long-term neuropathy” (score 19.1). The least important factors were: “My loved ones support treatment discontinuation” (score 5.2) and “Patients like me support treatment discontinuation” (score 3.3). “Relieving current neuropathy symptoms” (score 16.5) and “My oncologist supports treatment discontinuation” (score 13.3) were modestly important. Table 3 presents the importance of each factor in descending order (from most important to least important). BWS results were highly correlated with scale ratings in the familiarization exercise (Spearman’s *ρ* =. 0.79, *p*-value = 0.0410).

**Table 3:** Relative importance of factors influencing the decision to discontinue treatment (*n* = 189)

Factors	Coefficient^a^	SE	*P*-value	Importance score^b^
Having another cancer treatment option	0.96	0.06	< 0.001	23.47
Understanding the risk of treatment discontinuation	0.61	0.06	< 0.001	19.17
Reducing risk of long-term neuropathy	0.60	0.06	< 0.001	19.05
Relieving current neuropathy symptoms	0.38	0.06	< 0.001	16.52
My oncologist supports treatment discontinuation	0.07	0.05	0.202	13.28
My loved ones support treatment discontinuation	− 1.07	0.06	< 0.001	5.17
Patients like me support treatment discontinuation	− 1.55	0.07	< 0.001	3.33

^a^Due to effect coding, the sum of all coefficients equals zero, meaning positive values reflect factors chosen more often as important, while negative values reflect factors chosen less often

^b^Importance scores calculated by rescaling coefficients from conditional logit that sums up to 100, where higher scores indicate higher importance

*SE*, standard error

## Discussion

Given that about a third of patients with breast cancer have their treatment discontinued due to CIPN [45], understanding patients’ priorities when deciding about treatment discontinuation may improve clinician-patient communication and treatment outcomes. In our study, patients identified having another cancer treatment option as the most important factor, followed by understanding the risk of treatment discontinuation, and reducing risk of long-term neuropathy. Factors related to support from loved ones or patients with similar experiences were the least important when deciding to discontinue treatment due to CIPN. The high correlation between these findings and ratings obtained during the familiarization with the factors exercise lend validity to the study.

The two most important factors describe how the decision to switch or discontinue treatment due to CIPN may affect survival. Prioritizing survival was expected, as previous qualitative studies found women with CIPN to be reluctant to discontinue treatment due to fear of cancer recurrence or progression [16, 17]. However, in a few instances, women reported tolerating CIPN symptoms until physical function was significantly affected [17], suggesting that priorities may change with time and symptom experience [26]. In other qualitative studies, women with mBC were aware of their incurable condition but remained hopeful for a cure [46]. They acknowledged the severity of their cancer and the explicit threat of death [47]. This awareness and hope led them to continue palliative chemotherapy, accepting its side effects, including CIPN, as a necessary tradeoff for potential treatment benefits [48]. Our findings are consistent with four of six preference elicitation studies among patients with mBC who prioritized overall or progression-free survival over other treatment attributes [49–52]. Conversely, the other two studies found that patients with mBC prioritized quality of life attributes over treatment effectiveness [53, 54]. Differences in results may be due to variations in sample characteristics, the attributes and levels presented, and the decision context of the experiment. Further heterogeneity analysis, both observed and latent, is essential to discern preference differences based on clinical factors (e.g., CIPN severity, time since diagnosis) and patient characteristics (e.g., age, educational level) in a more extensive sample.

Patients in our study distinguished between short-term and long-term CIPN when contemplating treatment discontinuation. The importance placed on reducing the risk of long-term CIPN over relieving current CIPN symptoms aligns with findings from previous research. For example, a conjoint-analysis survey elicited preferences for taxane treatment among patients with early-stage breast cancer, revealing that patients shifted their preferences away from taxanes when CIPN levels were described as severe or irreversible [55]. Similarly, in a standard gamble technique study, women of all stages of breast cancer were willing to risk a 30.6% chance of mortality to avoid experiencing moderate to severe sensory CIPN for the remainder of their lives [56]. In a recent descriptive study, patients with CIPN expressed willingness to discontinue treatment for less severe CIPN if they were informed that their symptoms would become permanent [57]. Thus, it is evident that the duration of symptoms influences treatment decisions. Given that CIPN symptoms persist in 40 to 60% of patients 3 years following treatment [58], clinicians should be diligent in addressing the potential for long-lasting, permanent CIPN when discussing treatment discontinuation with their patients. Unfortunately, predicting whether CIPN will be persistent or irreversible is not possible, making it challenging to communicate this risk to patients [58]. Therefore, developing predictive tools for persistent CIPN becomes critical for informed decision-making.

In prior research, patients with CIPN reported relying on their oncologist [16], or loved ones to tell them how much CIPN they should endure [16], and seeking advice on how to manage their CIPN symptoms from support groups [16]. However, when measured and compared to other factors that influence decision-making, our results indicate that support from the oncologist, loved ones, or other patients was the least important. We posit that, in practice, patients may be unaware of the availability of alternative drugs, the risks associated with discontinuation, and the risks of CIPN permanence [17, 26, 59]. Patients often rely on their oncologists to possess this information and make the optimal decision on their behalf [16]. Consequently, if patients were placed in a hypothetical scenario where they have access to such information, it is unsurprising that the level of support from their oncologists becomes less influential. This underscores the necessity for tools that empower patients with information, enabling them to play a more active role in decision-making, rather than having to defer entirely to their clinicians [18, 60]. Examples of such tools are prompt sheets—sets of questions used by patients to acquire information during consultations [60]—and decision aids, which simplify medical evidence into a format accessible to patients, offering information on options, benefits, and risks, while also helping to clarify personal values [60].

Data from this study can inform clinical care by providing insight into factors that matter most to patients with mBC when deciding to discontinue treatment due to CIPN. Previous qualitative research has identified enablers (e.g., knowledge of CIPN as a side effect of treatment, positive relationship with the clinician, sufficient appointment time) and deterrents (e.g., fear of treatment discontinuation, lack of knowledge of long-term CIPN) of communication around CIPN between patients and clinicians [17]. It is, therefore, important for oncology clinicians to build trust with their patients by listening to and incorporating their priorities in decision-making. This not only improves patient-clinician relationship but also enhances treatment outcomes and patient satisfaction [13]. Our findings suggest that women with mBC are primarily influenced by having the option to switch to less neurotoxic treatment alternatives, understanding the potential impact that treatment discontinuation has on cancer progression, and minimizing the risk of long-term CIPN. As a result, it may be sufficient for clinicians to regularly inquire about which of these aspects matter the most to patients and educate them about their options. Further research is necessary to develop decision aid tools, such as informational sheets or pamphlets, that can offer valuable information without significantly extending consultation times [61].

A key strength of our study is its focus on a well-defined patient population and decision-making context. While reductions or delays are common strategies to manage CIPN progression, we specifically examined the discontinuation of neurotoxic treatment, as it represents a distinct clinical and emotional experience. The factors influencing this decision and how patients weigh them may differ from those affecting dose modifications. Additionally, our study focused on patients with mBC, whose treatment priorities differ from those with early-stage disease. While early-stage patients typically undergo treatment with a defined endpoint aimed at cure, those with mBC receive therapy indefinitely, balancing disease control with quality of life. Differences in prognosis, side effect tolerance, and treatment goals may shape how patients approach discontinuation decisions. Given these distinctions, our findings are specific to patients with mBC considering neurotoxic treatment discontinuation and cannot be generalizable to other settings.

This study has some limitations. First, the findings are susceptible to common biases in survey research. For example, 65% of respondents in our study had a bachelor’s degree or higher, suggesting potential self-selection and voluntary response biases. Given that BWS requires higher cognitive engagement than standard surveys, individuals with higher education levels may have been more likely to participate. Additionally, patients who had stronger opinions or greater interest in CIPN may have been more inclined to respond. No data were collected of individuals who declined participation; thus, comparing the characteristics of respondents and non-respondents was not possible. Second, while our findings reflect the average priorities of the study participants, individual patient choices may differ in real-world settings. Further research is needed to explore differences in ranking based on patient (e.g., age) and clinical (e.g., CIPN severity) characteristics. A latent class analysis could help identify subgroups with distinct decision-making patterns. Our data seemed to indicate that there were no measurable latent classes, likely due to sample size limitations. Third, although BWS is less cognitively demanding than a discrete choice experiment, the absence of CIPN severity and duration levels may have influenced responses. Patient priorities might differ if the symptoms were described as severe or permanent. Fourth, due to the cross-sectional design of the study, we were unable to capture maximum CIPN severity during treatment, as accurately recalling symptom severity at the time of decision-making can be challenging for some respondents. Finally, decision-making among patients with mBC is complex as they often navigate multiple treatment regimens and contend with competing risks. The decision to specifically focus on CIPN was deliberate but may not fully encompass real-world decision-making scenarios, which involve other clinical, financial, and emotional aspects.

## Conclusion

When faced with the decision to discontinue treatment due to CIPN, patients with mBC chose availability of less neurotoxic treatment options and understanding the potential impact of treatment discontinuation on cancer progression as the most important factors. Patients also highly valued minimizing the risk of long-term neuropathy, emphasizing the importance of addressing this concern. In practice, oncologists could elicit patient priorities by asking what matters most to them, enabling informed decision-making and facilitating discussions between oncologists and their patients to select a tailored treatment plan that aligns with patients’ desired outcomes. By identifying the most important factors influencing treatment discontinuation due to CIPN, our findings provide a foundation for conceptualizing and informing the development of a CIPN decision aid to streamline the decision-making process and improve the quality of care for patients with cancer experiencing CIPN.

## Supplementary Information

Below is the link to the electronic supplementary material.

Supplementary file1 (DOCX 48.8 KB)

## Data Availability

The datasets during and/or analyzed during the current study are available from the corresponding author on reasonable request.
