# Supplementary material for: Factors influencing the decision to discontinue treatment due to chemotherapy-induced peripheral neuropathy among patients with metastatic breast cancer: a best–worst scaling
Source: Support Care Cancer. 2025 May 10;33(6):467. doi: 10.1007/s00520-025-09508-4 (PMC12065726; doi:10.1007/s00520-025-09508-4)
Supplement: Supplementary file 1 — Supplementary file1 (DOCX 48.8 KB) [file 520_2025_9508_MOESM1_ESM.docx]

**Appendix A: Study Survey**

Thank you for your interest in our study. Before we proceed, we need to verify that you are eligible to participate. Please answer the following questions:

Are you 18 years or older?

- Yes
- No

Are you a woman?

- Yes
- No

Do you live in the US?

- Yes
- No

Has a doctor ever told you that you have metastatic breast cancer?

- Yes
- No

What side effects from cancer treatment have you experienced? (Select all that apply)

- Hair loss
- Nausea/vomiting
- Neuropathy (experience of tingling, numbness, muscle weakness, or pain in the hands and feet)
- Neutropenia (a drop in white blood cells count)
- Congestive heart failure (damage to the heart muscle)

**Study title:** Priorities and Preferences for Chemotherapy Discontinuation due to Chemotherapy Induced Peripheral Neuropathy Among Women with Metastatic Breast Cancer

**Purpose of study:** This survey explores your priorities and preferences when discontinuing **chemotherapy treatment (oral or infusion)** because of chemotherapy-induced peripheral neuropathy, or simply neuropathy. Neuropathy is nerve damage that can cause various symptoms in your hands and feet, including pain, numbness, and balance problems. It can make daily tasks difficult and increase your risk of falling. Because there is no cure for neuropathy, your doctor might suggest discontinuing treatment to prevent neuropathy from getting worse.

**Study procedure:** The survey comprises four sections. The first section will ask questions related to your disease, the second section will focus on your priorities, and the final section will gather information about you. The survey will take approximately 30 minutes to complete. Because we will ask about neuropathy and its effects on the body, some questions may make you feel uncomfortable. If you wish, you can take a break and come back later using the same device. If you close the survey and reopen it in the same browser, you will be able to continue from where you left off. Please note that you have **1 week** from this day to finish. After that, your response will be recorded as incomplete.

**Voluntary Participation:** This survey is **voluntary** and **anonymous**. You can stop taking the survey at any time. However, to receive compensation for your time ($50), you must complete **all of the questions**.

Do you agree to participate?

- Yes
- No

**Section 1: Medical history and neuropathy**

We will start by asking you some questions about your cancer and the neuropathy symptoms that you have experienced.

When were you diagnosed with metastatic breast cancer?

- Less than 1 year ago
- Between 1 and 5 years ago
- More than 5 years ago

Are you currently receiving treatment for metastatic breast cancer?

- Yes
- No

Which hormonal treatment **have you received or are you still receiving** for **metastatic breast cancer**? (Select all that apply)

- Tamoxifen (Nolvadex)
- Anastrozole (Arimidex)
- Goserelin (Zoldex)
- Letrozole (Femara)
- Leuprorelin (Lupron)
- Exemestane (Aromasin)
- Fulvestrant (Faslodex)
- Elacestrant (Orserdu)
- Triptorelin with Lupron (Trelstar)
- I have not received any hormonal therapy
- Other. Please specify: ____________________

Which treatment regimen are you **currently receiving** for **metastatic breast cancer**? (Select all that apply)

- AC-T (Adriamycin/Doxorbicin, Cyclophosphamide, and Paclitaxel/Taxol)
- AC-T (Adriamycin/Doxorubicin, Cyclophosphamide and Paclitaxel/Taxol with or without Herceptin/Trastuzumab (AC-TH) and Pertuzumab/Perjeta (AC-THP))
- AC-TC-Pembro (Adriamycin/Doxorubicin, Cyclophosphamide, Paclitaxel/Taxol, Carboplatin and Pembrolizumab)
- Affinitor (Everolimus)
- Capecitabine/Xeloda
- Enhertu or Fam-trastuzumab-deruxtecan
- Eribulin, Navelbine, Carboplatin, Cisplatin, Gemcitabine
- Kadcyla or TDM-1
- Neratinib
- Paclitaxel//Taxol or Docetaxel
- TC (Docetaxel and Cyclophosphamide)
- TCH (Taxotere/Docetaxel, Carboplatin, Herceptin/Trastuzumab) with or without Pertuzumab/Perjeta (TCHP)
- THP (Docetaxel or Paclitaxel plus Herceptin/Trastuzumab plus Pertuzumab/Perjeta) Tucatinib/Tukysa plus Capecitabine/Xeloda plus Trastuzumab/Herceptin
- Ibrance (Palbociclib)
- Piqray (Alpelisib)
- Kisqali (Ribociclib)
- Verzenio (Abemaciclib)
- I have not received any of these treatment regimens
- Other. Please specify: ____________________

Which treatment regimen have you **previously received** for **metastatic breast cancer**? (Select all that apply)

- AC-T (Adriamycin/Doxorbicin, Cyclophosphamide, and Paclitaxel/Taxol)
- AC-T (Adriamycin/Doxorubicin, Cyclophosphamide and Paclitaxel/Taxol with or without Herceptin/Trastuzumab (AC-TH) and Pertuzumab/Perjeta (AC-THP))
- AC-TC-Pembro (Adriamycin/Doxorubicin, Cyclophosphamide, Paclitaxel/Taxol, Carboplatin and Pembrolizumab)
- Affinitor (Everolimus)
- Capecitabine/Xeloda
- Enhertu or Fam-trastuzumab-deruxtecan
- Eribulin, Navelbine, Carboplatin, Cisplatin, Gemcitabine
- Everolimus (Affinitor)
- Kadcyla or TDM-1
- Neratinib
- Paclitaxel//Taxol or Docetaxel
- TC (Docetaxel and Cyclophosphamide)
- TCH (Taxotere/Docetaxel, Carboplatin, Herceptin/Trastuzumab) with or without Pertuzumab/Perjeta (TCHP)
- THP (Docetaxel or Paclitaxel plus Herceptin/Trastuzumab plus Pertuzumab/Perjeta)
- Tucatinib/Tukysa plus Capecitabine/Xeloda plus Trastuzumab/Herceptin
- Ibrance (Palbociclib)
- Piqray (Alpelisib)
- Kisqali (Ribociclib)
- Verzenio (Abemaciclib)
- I have not received any of these treatment regimens
- Other. Please specify: ____________________

Are you **currently** experiencing neuropathy (pain, tingling, numbness, or muscle weakness in the hands or feet) from your past or current chemotherapy treatment?

- Yes
- No

How many years has it been since you started experiencing neuropathy from chemotherapy treatment?

- Less than 1 year
- Between 1 and 5 years
- More than 5 years

In the past 7 days, what was the severity of numbness and tingling in your hands or feet at their worst?

- None
- Mild
- Moderate
- Severe
- Very severe

In the past 7 days, how much did numbness or tingling in your hands or feet interfere with your usual or daily activities?

- Not at all
- A little bit
- Somewhat
- Quite a bit
- Very much

Have you ever used medications such as Duloxetine (Cymbalta, Irenka), Pregabalin (Lyrica), Gabapentin (Neurontin), or any other medication to manage neuropathy?

- Yes
- No

Have medications such as Duloxetine (Cymbalta, Irenka), Pregabalin (Lyrica), Gabapentin (Neurontin), or any other medication, helped relieve your neuropathy symptoms?

- Yes
- Not sure
- No

Have you ever used non-drug strategies such as acupuncture, physical therapy, cryotherapy (e.g. frozen gloves and socks), supplements (e.g., vitamin B12), or any other non-drug strategies to manage neuropathy?

- Yes
- No

Have non-drug strategies such as acupuncture, physical therapy, cryotherapy (e.g. frozen gloves and socks), supplements (e.g., vitamin B12), or any other non-drug strategies, helped relieve your neuropathy symptoms?

- Yes
- Not sure
- No

Has your doctor ever recommended altering (reducing, delaying, or discontinuing) chemotherapy treatment due to neuropathy?

- Yes
- No

What changes were performed to your chemotherapy treatment because of neuropathy? (Select all that apply)

- Reduce dose
- Delay treatment
- Discontinue treatment and switch to another therapy

Do you think that chemotherapy treatment alteration improved your neuropathy symptoms?

- Yes
- No

Do you have any regrets about altering chemotherapy treatment because of neuropathy?

- Yes
- No

**Section 2: Understanding what factors influence patients’ decision to discontinue treatment because of neuropathy**

Doctors may recommend discontinuing chemotherapy treatment to prevent neuropathy from worsening. We would like to know how important the following things are to you when deciding to discontinue chemotherapy treatment because of neuropathy:

|  | Not Important | Important | Very Important |
| --- | --- | --- | --- |
| **Relieving current neuropathy symptoms**  Treatment discontinuation can help relieve the neuropathy symptoms that you are currently experiencing. | O | O | O |
| **Reducing risk of long-term neuropathy**  Treatment discontinuation can lower the risk of developing long-term neuropathy that lasts for years even after discontinuing the treatment that was causing neuropathy. | O | O | O |
| **Having another cancer treatment option**  Having the option to switch to another cancer treatment that doesn’t cause neuropathy but still helps fight off cancer. | O | O | O |
| **My oncologist supports treatment discontinuation**  Support from your oncologist to discontinue treatment. | O | O | O |
| **My loved ones support treatment discontinuation**  Support from loved ones, such as family, friends, or partners to discontinue treatment. | O | O | O |
| **Patients like me support treatment discontinuation**  Support from other patients who have undergone similar experiences and have made similar decisions. | O | O | O |

Consider the following situation: Sara is trying to decide, together with her oncologist, whether or not to discontinue chemotherapy treatment because of neuropathy. If you were Sara, what things would influence your decision to discontinue treatment because of neuropathy?

First, we will show you an example of what Sara chose, then in the next page, it will be your turn to choose.

**Here is the example:**

Sara was shown the table below and was asked to pick the **most** and **least** important thing to influence the decision to discontinue treatment because of neuropathy.

| Most Important  (Pick one) |  | Least important  (Pick one) |
| --- | --- | --- |
| O | Relieving current neuropathy symptoms | O |
| O | Having another cancer treatment option | O |
| O | Patients like me support treatment discontinuation | O |
| O | My oncologist supports treatment discontinuation | O |

After carefully considering all four things, Sara chose “Relieving current neuropathy symptoms” as the most important thing to influence her decision to discontinue treatment and “Having another cancer treatment option” as the least important thing to influence her decision to discontinue treatment.

**Now, it’s your turn:**

In the next pages, we will show you seven tables similar to the one you saw before with 4 things at a time. For each table, pick the **most** and **least** important thing to influence your decision to discontinue chemotherapy treatment because of neuropathy. **The options will be repeated in different combinations, your choices might stay the same or change.**

Remember, this is not a test and there are no right or wrong answers.

Please pick the **most** and **least** important thing to influence your decision to discontinue chemotherapy treatment because of neuropathy

| Most Important  (Pick one) |  | Least important  (Pick one) |
| --- | --- | --- |
| O | Relieving current neuropathy symptoms | O |
| O | Having another cancer treatment option | O |
| O | Patients like me support treatment discontinuation | O |
| O | My oncologist supports treatment discontinuation | O |

Please pick the **most** and **least** important thing to influence your decision to discontinue chemotherapy treatment because of neuropathy

| Most Important  (Pick one) |  | Least important  (Pick one) |
| --- | --- | --- |
| O | My loved ones support treatment discontinuation | O |
| O | Patients like me support treatment discontinuation | O |
| O | Reducing risk of long-term neuropathy | O |
| O | Having another cancer treatment option | O |

Please pick the **most** and **least** important thing to influence your decision to discontinue chemotherapy treatment because of neuropathy

| Most Important  (Pick one) |  | Least important  (Pick one) |
| --- | --- | --- |
| O | My oncologist supports treatment discontinuation | O |
| O | Reducing risk of long-term neuropathy | O |
| O | Relieving current neuropathy symptoms | O |
| O | My loved ones support treatment discontinuation | O |

Please pick the **most** and **least** important thing to influence your decision to discontinue chemotherapy treatment because of neuropathy

| Most Important  (Pick one) |  | Least important  (Pick one) |
| --- | --- | --- |
| O | Patients like me support treatment discontinuation | O |
| O | My oncologist supports treatment discontinuation | O |
| O | My loved ones support treatment discontinuation | O |
| O | Understanding the risk of treatment discontinuation | O |

Please pick the **most** and **least** important thing to influence your decision to discontinue chemotherapy treatment because of neuropathy

| Most Important  (Pick one) |  | Least important  (Pick one) |
| --- | --- | --- |
| O | Understanding the risk of treatment discontinuation | O |
| O | My loved ones support treatment discontinuation | O |
| O | Having another cancer treatment option | O |
| O | Relieving current neuropathy symptoms | O |

Please pick the **most** and **least** important thing to influence your decision to discontinue chemotherapy treatment because of neuropathy

| Most Important  (Pick one) |  | Least important  (Pick one) |
| --- | --- | --- |
| O | Having another cancer treatment option | O |
| O | Understanding the risk of treatment discontinuation | O |
| O | My oncologist supports treatment discontinuation | O |
| O | Reducing risk of long-term neuropathy | O |

Please pick the **most** and **least** important thing to influence your decision to discontinue chemotherapy treatment because of neuropathy

| Most Important  (Pick one) |  | Least important  (Pick one) |
| --- | --- | --- |
| O | Reducing risk of long-term neuropathy | O |
| O | Relieving current neuropathy symptoms | O |
| O | Understanding the risk of treatment discontinuation | O |
| O | Patients like me support treatment discontinuation | O |

**Section 3: Demographic and psychosocial characteristics**

Lastly, please answer the following questions about yourself.

What is your age? [Text-box]

What is your marital status?

- Single
- In a relationship

Do you have children who are younger than 18 years old?

- Yes
- No

Which of the following racial or ethnic groups best describes you?

- Black, non-Hispanic
- White, non-Hispanic
- Hispanic
- Other, non-Hispanic

What is the highest level of education you have completed?

- High school or less
- Some college
- Bachelor’s degree or higher

What state do you live in? [drop-down menu]

What is your household income?

- Less than 34,999
- 35,000–84,999
- More than 85,000
- Prefer not to say

These questions ask you about your ability to understand cancer-related medical information.

The normal range for hemoglobin for a male is 13.3 - 17.2 g/dL. Joe’s hemoglobin is 9.7 g/dL. Is Joe within the normal range?

- Yes
- No

A biopsy of a tumor is done to...

- Remove it
- Diagnose it
- Treat it

If a patient has stage 1 cancer, it means the cancer is...

- Localized
- In nearby organ
- In distant sites

The role of a physical therapist is to talk to a patient about emotional needs

- True
- False

A tumor is considered ‘‘inoperable’’ when it cannot be treated with...

- Radiation therapy
- Surgery
- Chemotherapy

Sally will get radiation therapy once a day, Monday through Friday. If Sally has therapy for 4 weeks, how many times will she get radiation therapy?

- 5
- 15
- 20

These questions ask about your relationship with the doctor who prescribed your chemotherapy. Indicate the extent you agree or disagree with the following statements.

| Statement | Strongly  disagree | Disagree | As much agree as  disagree | Agree | Strongly  agree |
| --- | --- | --- | --- | --- | --- |
| Your doctor is totally honest in telling you about all the different treatment options available for your condition | O | O | O | O | O |
| You think your doctor can handle any medical situation, even a very serious one | O | O | O | O | O |
| Your doctor listens with care and concern to all the problems you have | O | O | O | O | O |
| Your doctor will do whatever it takes to get you all the care you need | O | O | O | O | O |
| All in all, you have complete trust in your doctor | O | O | O | O | O |

| I worry about…. | Not at all | A little bit | Somewhat | Very much |
| --- | --- | --- | --- | --- |
| Future diagnostic tests | O | O | O | O |
| Another type of cancer | O | O | O | O |
| Dying | O | O | O | O |
| My health | O | O | O | O |
| My children’s health | O | O | O | O |

These questions ask you to indicate the degree to which you have fears about cancer recurrence and health using the 1 (not at all) to 4 (very much) scale.

Thank you for completing the survey!
